# Deep Learning‐Enabled Automated Quality Control for Liver MR Elastography: Initial Results

**DOI:** 10.1002/jmri.29490

**Published:** 2024-06-22

**Authors:** Heriberto A. Nieves‐Vazquez, Efe Ozkaya, Waiman Meinhold, Amine Geahchan, Octavia Bane, Jun Ueda, Bachir Taouli

**Affiliations:** ^1^ Department of Biomedical Engineering Georgia Institute of Technology Atlanta Georgia USA; ^2^ BioMedical Engineering and Imaging Institute Icahn School of Medicine Mount Sinai New York New York USA; ^3^ Department of Diagnostic, Molecular and Interventional Radiology Icahn School of Medicine at Mount Sinai New York New York USA; ^4^ School of Mechanical Engineering Georgia Institute of Technology Atlanta Georgia USA

**Keywords:** deep learning, liver stiffness, magnetic resonance elastography, image quality control

## Abstract

**Background:**

Several factors can impair image quality and reliability of liver magnetic resonance elastography (MRE), such as inadequate driver positioning, insufficient wave propagation and patient‐related factors.

**Purpose:**

To report initial results on automatic classification of liver MRE image quality using various deep learning (DL) architectures.

**Study Type:**

Retrospective, single center, IRB‐approved human study.

**Population:**

Ninety patients (male = 51, mean age 52.8 ± 14.1 years).

**Field Strengths/Sequences:**

1.5 T and 3 T MRI, 2D GRE, and 2D SE‐EPI.

**Assessment:**

The curated dataset was comprised of 914 slices obtained from 149 MRE exams in 90 patients. Two independent observers examined the confidence map overlaid elastograms (CMOEs) for liver stiffness measurement and assigned a quality score (non‐diagnostic vs. diagnostic) for each slice. Several DL architectures (ResNet18, ResNet34, ResNet50, SqueezeNet, and MobileNetV2) for binary quality classification of individual CMOE slice inputs were evaluated, using an 8‐fold stratified cross‐validation (800 slices) and a test dataset (114 slices). A majority vote ensemble combining the models' predictions of the highest‐performing architecture was evaluated.

**Statistical Test:**

The inter‐observer agreement and the agreement between DL models and one observer were assessed using Cohen's unweighted Kappa coefficient. Accuracy, precision, and recall of the cross‐validation and the ensemble were calculated for the test dataset.

**Results:**

The average accuracy across the eight models trained using each architecture ranged from 0.692 to 0.851 for the test dataset. The ensemble of the best performing architecture (SqueezeNet) yielded an accuracy of 0.921. The inter‐observer agreement was excellent (Kappa 0.896 [95% CI 0.845–0.947]). The agreement between observer 1 and the predictions of each SqueezeNet model was slight to almost perfect (Kappa range: 0.197–0.831) and almost perfect for the ensemble (Kappa: 0.833).

**Conclusion:**

Our initial study demonstrates an automated DL‐based approach for classifying liver 2D MRE diagnostic quality with an average accuracy of 0.851 (range 0.675–0.921) across the SqueezeNet models.

**Evidence Level:**

4

**Technical Efficacy:**

Stage 1

Magnetic resonance elastography (MRE) is a complex imaging method that uses motion encoding to measure and display the mechanical characteristics of tissues within the body. It combines magnetic resonance imaging (MRI) with mechanical vibrations to generate detailed maps of tissue stiffness. Through the generation of these maps, known as elastograms, MRE gathers data on tissue stiffness and is commonly used to noninvasively assess the stage of liver fibrosis, which allows the evaluation of the severity of liver disease and informs clinical management.^1^, ^2^

During the MRE acquisition, two datasets of wrapped phase images with opposite polarities are created. By subtracting the unwrapped datasets from each other, the static background information is eliminated, preserving only the propagating wave information.^3^ In addition to phase and wave images, magnitude images, confidence maps, elastograms, and confidence map overlaid elastograms (CMOEs) are also created.^4^

In MRE, patient‐ or hardware‐related factors can lead to failure, reported to be as high as 15.3% with 2D gradient‐echo (GRE) sequence at 3 T.^5^, ^6^, ^7^, ^8^, ^9^ So far, determining the failure of MRE has relied on visual inspection,^7^, ^9^, ^10^ which can be time‐consuming and challenging for inexperienced operators. This process could result in unnecessary additional table time, underscoring the need for a streamlined approach.

Automated artifact detection has been described for various MRI applications, such as identifying wrap‐around and Gibbs ringing artifacts using explainable class activation map (CAM)‐enabled convolutional neural networks (CNNs),^11^ and identifying motion, chemical‐shift, and radio‐frequency artifacts using a CNN ensemble.^12^ Yet, to the best of our knowledge, artifact detection and correction strategies,^13^, ^14^ and quality control methods have not been described for 2D liver MRE using confidence map overlaid elastograms. Such a method that can detect a non‐diagnostic exam in real time can improve the current MRE workflow by decreasing the quality assessment time from minutes to seconds.

The objective of this initial study was to automatically classify liver MRE image quality using various DL models.

## Materials and Methods

### Study Population

This was a retrospective single‐center study, approved by the Institutional Review Board with a patient consent exemption. To identify suitable patient records, a query search was conducted using keywords such as “elastography” and “MRE,” within the period from January 1, 2018, to December 31, 2022, utilizing the DICOM database within BioMedical Engineering and Imaging Institute, Icahn School of Medicine Mount Sinai, New York, NY, USA. Our inclusion criteria were: 1) consecutive patients who underwent MRI and MRE outside a clinical trial, 2) available grayscale CMOEs, which were required for training our models uniformly with a single channel input. Our exclusion criteria were: 1) patients who underwent MRE without grayscale CMOEs.

Our query search yielded a total of 1372 MRE exams, of which 149 MRE exams having gray‐scale CMOEs were manually chosen without any consideration given to BMI. The selection was performed with the aim to overrepresent failed MRE cases to create a class‐balanced dataset for DL training, comprising 914 CMOE slices from a total of 90 patients (51 males, mean age of 52.8 ± 14.1 years) with mean body mass index (BMI) of 33.3 ± 8.0 (range: 20.1–51.5) who underwent 2D liver MRE using 1.5 T or 3 T.

### MRE Acquisition

MRE exams with two different sequences were collected, including a dataset with a 2D spin‐echo echo‐planar imaging (SE‐EPI) sequence acquired at 1.5 T (MAGNETOM Aera, Siemens Healthineers, N = 51) or 3 T (MAGNETOM Skyra, Siemens Healthineers, N = 25); and a dataset with a 2D GRE sequence acquired at 1.5 T (MAGNETOM Aera, N = 13) or 3 T (MAGNETOM Skyra, N = 1). Both MRE protocols operated at a vibration frequency of 60 Hz using a Resoundant system (Rochester, MN, USA). Scan parameters are described in Table 1.

**TABLE 1:** 2D MRE Acquisition Parameters Obtained on Siemens Systems

Scanner	1.5 T MAGNETOM Aera		3 T MAGNETOM Skyra	
Sequence Type	SE‐EPI	GRE	SE‐EPI	GRE
Orientation	Axial	Axial	Axial	Axial
TR (msec)	1500, 2000	50	1000, 1400	50
TE (msec)	41, 48, 52	20.7	48–50	22
Flip angle (°)	90	20, 30	90	25
FOV (mm^2^)	362 × 400–453 × 500	407 × 450–440 × 440	362 × 400–453 × 500	400 × 400
Matrix	232 × 256, 256 × 256	232 × 256, 256 × 256	232 × 256, 256 × 256	256 × 128
Slice thickness (mm)	3, 5, 8	10	8	8
Number of slices	4–10	4–8	4–6	4–8
Acceleration (GRAPPA)	*R* = 2	*R* = 2	*R* = 2	*R* = 2

Comma denotes separate values and en dash indicates range of values. FOV = field of view; GRE = gradient echo; MRE = magnetic resonance elastography; SE‐EPI = spin‐echo echo‐planar imaging; TE = echo time; TR = repetition time; GRAPPA = generalized autocalibrating partially parallel acquisitions.

### Dataset Processing

Initially, the axial CMOEs were de‐identified and preserved in DICOM format. Subsequently, these CMOEs in grayscale format were normalized to a range of [0, 255]. Next, they were resized to dimensions of 224 × 224. Finally, each individual slice was saved as a NumPy array for convenient utilization within the PyTorch framework.

### Dataset Labeling

The CMOEs within the entire dataset were labeled based on the percentage of the area of liver parenchyma with 95% or higher confidence as required in clinical practice.^4^, ^9^ An observer (observer 1, E.O., a physicist with 2 years of experience in MRE) evaluated each CMOE, and if needed the other MRE image outputs corresponding to that acquisition such as magnitude and phase/wave, to determine whether the MRE exam failed or not, assigning the quality of the image acquisition as diagnostic or non‐diagnostic, as follows: label 0 (non‐diagnostic quality, <25% area of confidence in the liver parenchyma), and label 1 (diagnostic quality, ≥25% area of confidence in the liver parenchyma).^9^ Another observer (observer 2, A.G., a radiologist with 1 year of experience in MRE) reviewed approximately 294 slices from the dataset to evaluate inter‐observer agreement.

### Classification Model

**FIGURE 1:**
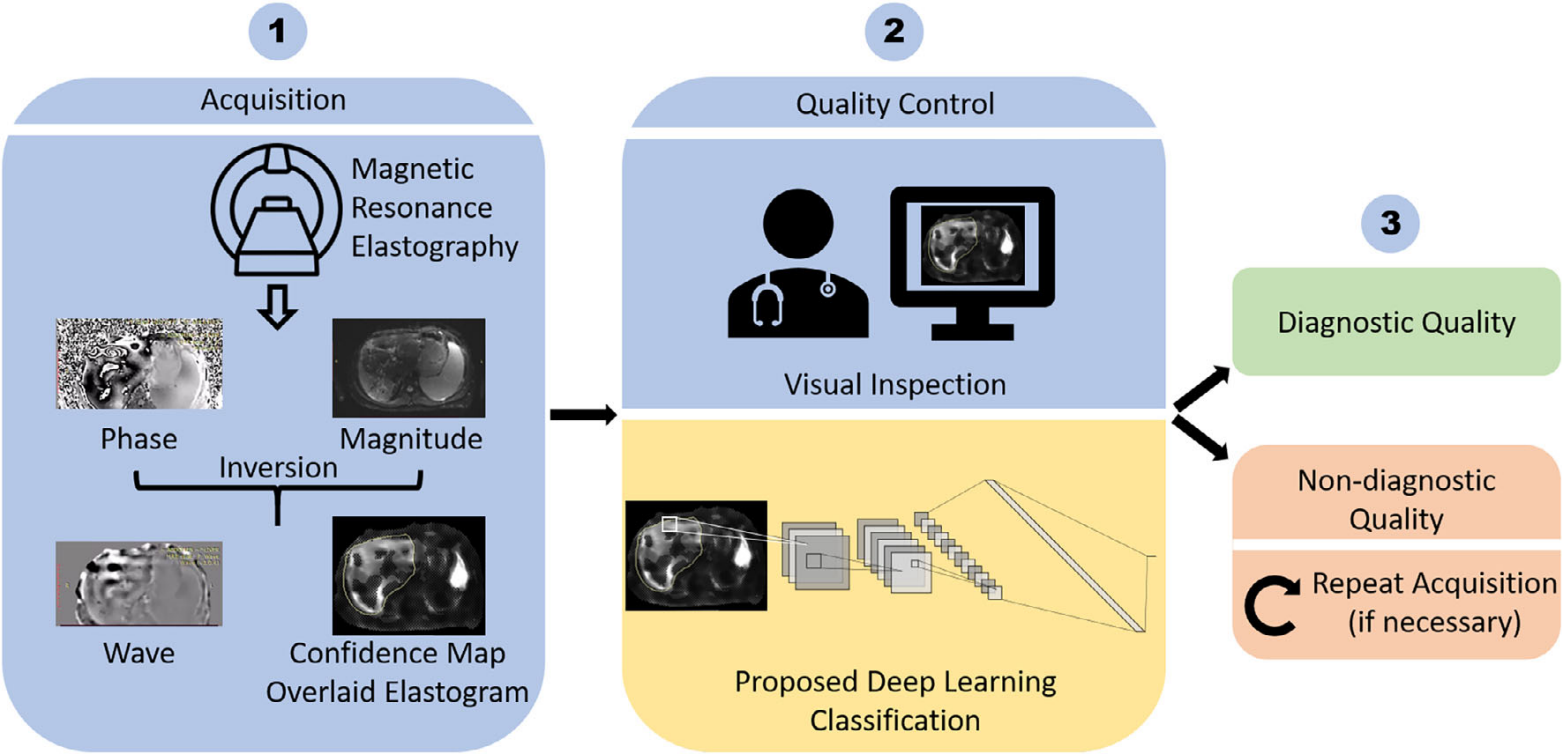
Comparison of the workflow between the proposed deep learning‐based image quality classification and the traditional visual inspection.

**FIGURE 2:**
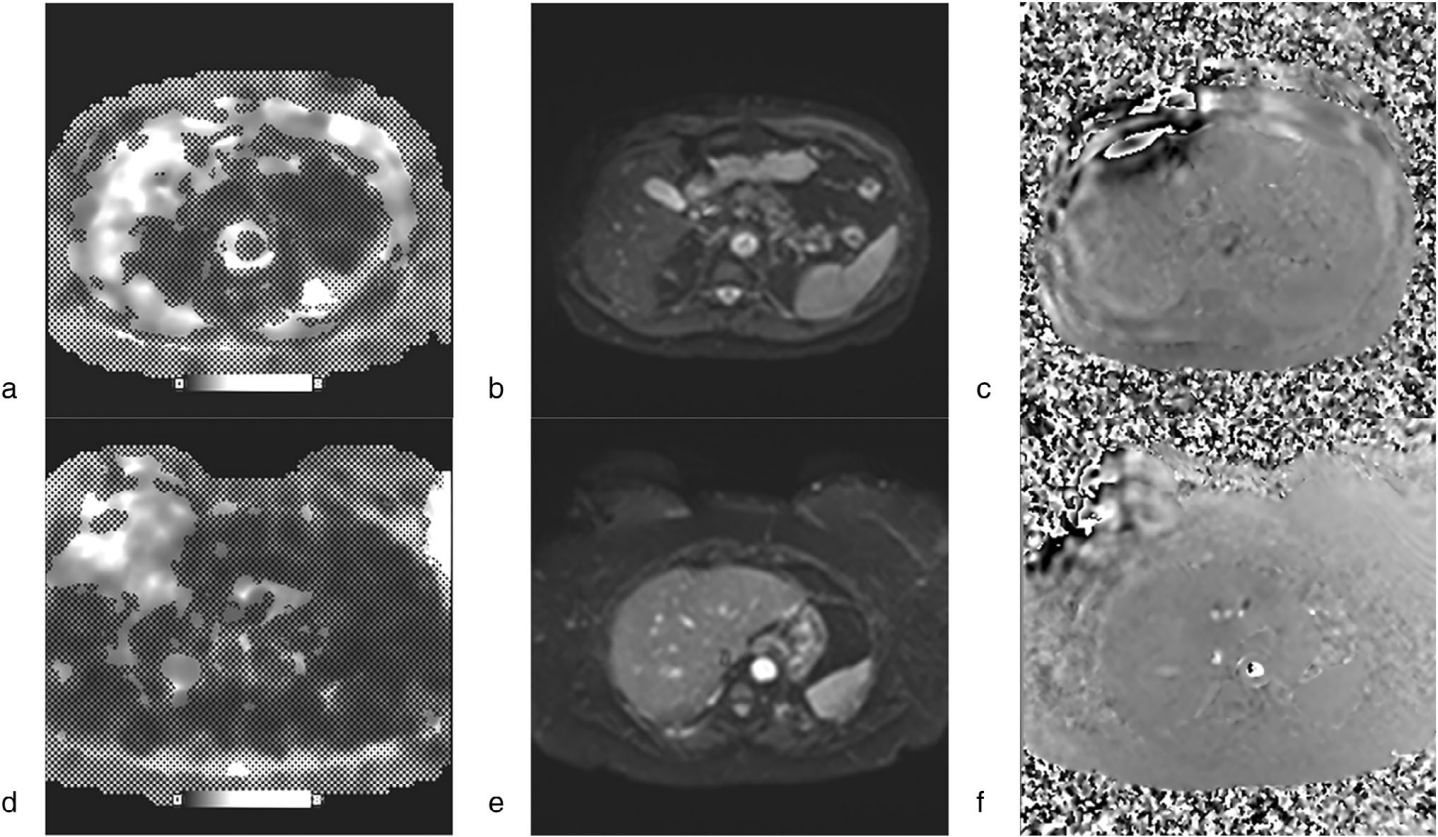
Examples of curated liver MRE datasets, including a diagnostic quality confidence map overlaid elastogram (CMOE) (**a**) obtained using 2D SE‐EPI at 3 T (top row) and a non‐diagnostic quality CMOE (**d**) obtained using 2D SE‐EPI at 1.5 T (bottom row). The corresponding magnitude (**b**, **e**) and phase (**c**, **f**) images are also shown for each CMOE slice.

The objective of the present work was to enhance the MRE quality evaluation process by evaluating various CNNs to obtain a binary classification of CMOEs, determining whether they provided diagnostic or non‐diagnostic quality for liver stiffness measurement. For this study, we assessed well‐established DL architectures such as ResNet18, ResNet34, ResNet50, SqueezeNet, and MobileNetV2 in PyTorch^15^, ^16^, ^17^, ^18^, ^19^, ^20^ (Fig. 1). We chose these architectures because they are designed to handle 2D images with dimensions of 224 × 224 as inputs. Additionally, we customized the input convolutional layer of each architecture to accept an individual grayscale CMOE slice as input and adjusted the output layer of each architecture for binary classification purposes (Fig. 2). The number of trainable parameters for each architecture is 11.2M (ResNet18), 21.3M (ResNet34), 23.5M (ResNet50), 2.2M (MobileNetV2), 0.7M (SqueezeNet). During the model training, an Adam optimizer was used, along with a cross‐entropy loss function to be minimized (referred to as CrossEntropyLoss in PyTorch) (Equation 1) where i∈{0,1} is the class index, $t_{i}$ is a class value either 0 for non‐diagnostic quality and 1 for diagnostic quality, and $p_{i}$ is the probability of the respective class outputted by the CNN such that $\sum_{i=0}^{1}p_{i}=1$.

(1) $$\text{Loss}=‐\sum_{i=1}^{2}t_{i}\log\left(p_{i}\right).$$

To verify each architecture's performance and generalizability, an 8‐fold stratified cross‐validation was performed such that 7 folds, comprising 700 CMOE slices, were used for training and the final fold of 100 slices was used for validation. Each fold maintained the same diagnostic class balance (ratio of diagnostic slices to total number of slices) to mitigate biases during training, based on the reference standard for image quality evaluation reported in the Results. The remaining 114 slices (12.5%) of the dataset were used solely for testing and remained constant across all iterations. Training spanned 30 epochs with a batch size of 32 and a learning rate of 0.0001.^21^ Training/evaluation times for the iterations during the cross‐validation using an iMac (macOS Ventura 13.2, Processor 3.3 GHz 6‐Core Intel Core i5, Memory 8 GB 2667 MHz DDR4) are given in Table 3 for each architecture.

### Statistical Analysis

The image quality labeling from observer 1 was used as the reference standard for model training and evaluation. Training and validation accuracy (Equation 2) and loss (Equation 1) were calculated for all iterations of the 8‐fold cross‐validation. The accuracy, precision (Equation 3), and recall (Equation 4) of every trained model were also evaluated using the test dataset. To measure inter‐observer agreement and the agreement between the predictions of all DL models and observer 1, Cohen's unweighted Kappa coefficients were calculated.^22^ Additionally, an ensemble decision‐making approach in which the majority vote or mode of the test predictions across all iterations of the highest‐performing architecture was evaluated using the test dataset. The same accuracy, precision, recall, and inter‐observer agreement metrics were applied to the ensemble for comparison with individual folds.

(2) $$\text{Accuracy}=\frac{\mathrm{TP}+\mathrm{TN}}{\mathrm{TP}+\mathrm{FP}+\mathrm{TN}+\mathrm{FN}},$$

(3) $$\text{Precision}=\frac{\mathrm{TP}}{\mathrm{TP}+\mathrm{FP}},$$

(4) $$\text{Recall}=\frac{\mathrm{TP}}{\mathrm{TP}+\mathrm{FN}},$$

where FN, FP, TN, and TP are false negatives, false positives, true negatives, and true positives.

## Results

### Reference Standard

The curated dataset of 914 slices consisted of 574 slices (62.8%) of diagnostic quality, and 340 slices (37.2%) of non‐diagnostic quality from a total of 149 MRE acquisitions, including 56 failed MRE acquisitions. The technical failure rate was high due to the intentional inclusion of failed MRE datasets for training purposes. The stratified cross‐validation maintained a diagnostic class balance of 63.0% (ratio of diagnostic slices to total number of slices) in each of the folds, and the test dataset had a diagnostic class balance of 61.4%. The number of slices acquired using the 1.5 T system was 718 (78.5% of all slices), with 478 slices (66.5% of 1.5 T slices) labeled diagnostic. Similarly, the number of slices acquired using the 3 T system was 196 (21.5% of all slices) with 96 slices (49.0% of 3 T slices) labeled diagnostic. As for the sequences used for the acquisition, 838 slices (91.7% of all slices) were acquired using EPI, with 513 slices (61.2% of EPI slices) labeled as diagnostic. A total of 76 slices (8.3% of all slices) were acquired using GRE where 61 slices (80.3% of GRE slices) were labeled as diagnostic. The Cohen's unweighted Kappa coefficient between the two observers was 0.896 [95% CI: 0.845–0.947], indicating almost perfect agreement.

### DL Model Performance

**FIGURE 3:**
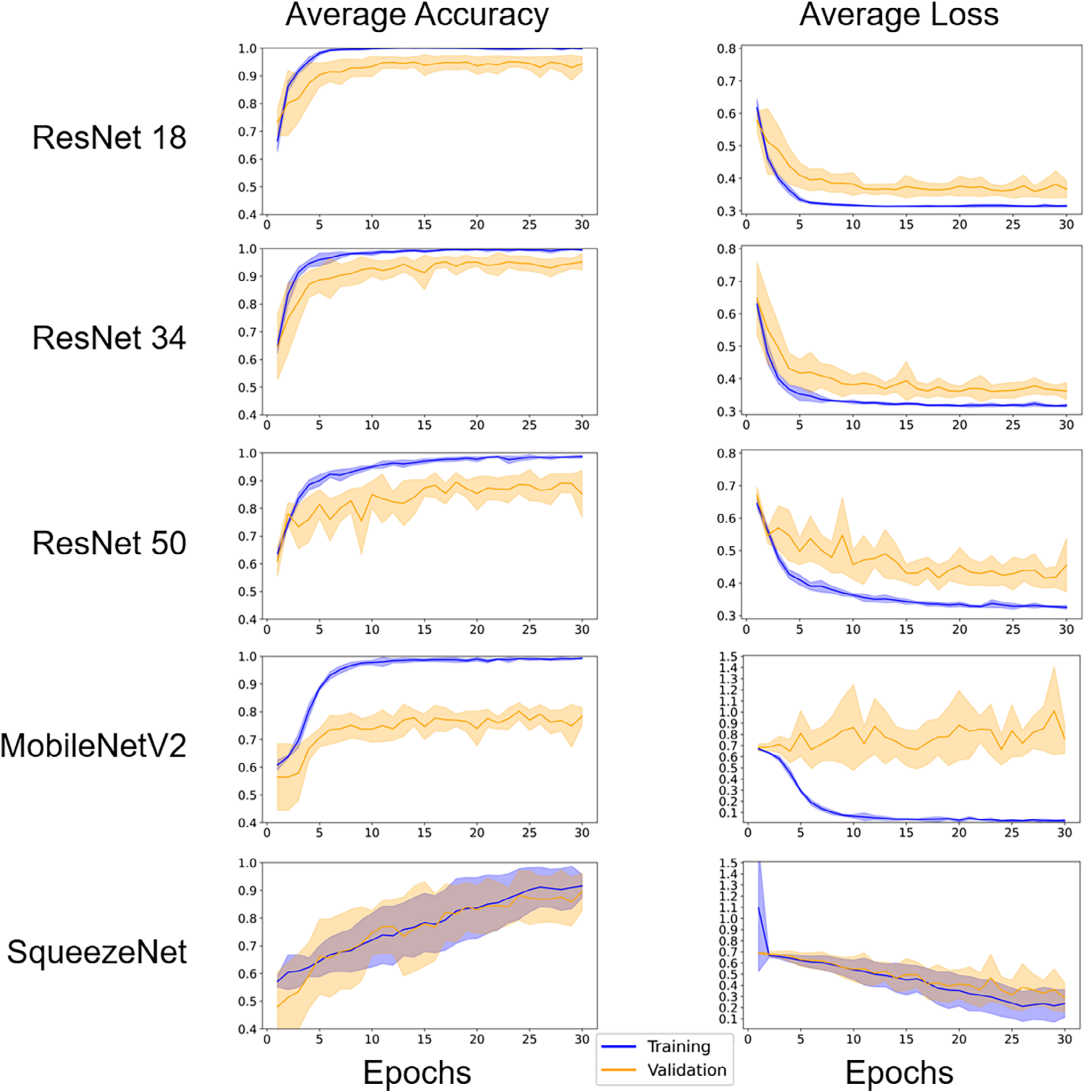
Training and validation accuracies and losses for each DL model across cross‐validation iterations. For each iteration, the training and validation datasets consisted of 700 and 100 slices, respectively. The solid line represents the average accuracy or loss across all eight iterations of the cross‐validation and the band represents the corresponding standard deviation.

The training/validation accuracy and loss averages with their standard deviations during the 30 epochs of training across all cross‐validation iterations for each architecture are shown in Fig. 3. Training for 30 epochs was found to yield optimal test accuracy performances, with little to no improvement occurring at higher epochs, and overfitting increasing at 35 epochs except for the SqueezeNet model. The average accuracies ranged from 0.692 to 0.851 using the test set (Table 2). The average training, validation, and test accuracies as well as test precision and test recall calculated from each trained model during the cross‐validations are shown in Table 3. The highest average accuracy (0.851), average precision (0.880), and average recall (0.713) were achieved across the SqueezeNet models. Therefore, we considered the SqueezeNet as the highest performing architecture in our study.

**TABLE 2:** Architecture‐Specific Trainable Parameters and Average Accuracies for the Training, Validation, and Test Datasets

Architecture (Number of Trainable Parameters)	Training Dataset (N^a^ = 700, 76.6%)	Validation Dataset (N^a^ = 100, 10.9%)	Test Dataset (N^a^ = 114, 12.5%)			
	Average Accuracy	Average Accuracy	Average Accuracy	Average Precision	Average Recall	Kappa Range
ResNet18 (11.2M)	**0.998**	0.944	0.798	0.839	0.594	[0.304–0.661]
ResNet34 (21.3M)	0.996	**0.953**	0.802	0.849	0.605	[0.403–0.646]
ResNet50 (23.5M)	0.987	0.853	0.739	0.756	0.534	[0.203–0.760]
MobileNetV2 (2.2M)	0.993	0.784	0.692	0.672	0.455	[0.225–0.393]
SqueezeNet (0.7M)	0.916	0.895	**0.851**	**0.880**	**0.713**	**[0.197–0.831]**

Average precision, recall, and agreement between each architectural model and reference standard (Cohen's unweighted Kappa coefficient) also provided for the test dataset. All metrics were calculated after 30 epochs of training. The highest value for each metric is highlighted in bold.

^a^ Number of slices.

**TABLE 3:** Duration of Iterations, Test Accuracy, and Agreement Between Each Model and Reference Standard (Cohen's Unweighted Kappa Coefficient) Using the Test Dataset

Architecture	Iteration	Duration (Sec)	Test Accuracy	Kappa Coefficient [95% CI]
ResNet18	1	2849.27	0.816	0.596 [0.443–0.749]
	2	2839.30	0.789	0.524 [0.365–0.683]
	3	2854.78	0.781	0.510 [0.349–0.672]
	4	2814.98	**0.842**	**0.661 [0.519–0.804]**
	5	2804.54	0.842	0.649 [0.505–0.793]
	6	2814.92	0.711	0.304 [0.149–0.459]
	7	2808.40	0.807	0.579 [0.424–0.734]
	8	2830.60	0.798	0.554 [0.396–0.711]
	Average	2827.09	0.798	
ResNet34	1	5118.22	0.789	0.524 [0.365–0.683]
	2	5110.68	0.825	0.607 [0.457–0.756]
	3	5112.63	0.798	0.546 [0.389–0.703]
	4	5112.18	0.833	0.631 [0.484–0.778]
	5	5125.35	0.719	0.403 [0.230–0.576]
	6	5104.48	0.798	0.541 [0.385–0.698]
	7	5102.54	0.816	0.596 [0.443–0.749]
	8	5101.98	**0.842**	**0.646 [0.502–0.790]**
	Average	5111.01	0.803	
ResNet50	1	8650.14	0.728	0.382 [0.212–0.552]
	2	8118.78	0.667	0.203 [0.044–0.361]
	3	8908.69	0.798	0.580 [0.427–0.732]
	4	7698.00	0.667	0.171 [0.041–0.300]
	5	7702.88	0.746	0.400 [0.241–0.559]
	6	7723.27	0.711	0.342 [0.170–0.515]
	7	7676.90	0.711	0.456 [0.318–0.594]
	8	7646.30	**0.886**	**0.760 [0.638–0.883]**
	Average	8015.62	0.739	
MobileNetV2	1	2641.32	0.684	0.339 [0.163–0.515]
	2	2639.84	**0.728**	**0.393 [0.221–0.565]**
	3	2563.58	0.675	0.262 [0.087–0.438]
	4	2530.83	0.675	0.346 [0.176–0.515]
	5	2539.81	0.667	0.225 [0.055–0.395]
	6	2514.16	0.667	0.253 [0.074–0.431]
	7	2530.47	0.719	0.347 [0.181–0.514]
	8	2559.55	0.719	0.328 [0.172–0.485]
	Average	2564.95	0.692	
SqueezeNet	1	2131.66	0.904	0.796 [0.681–0.910]
	2	2121.37	**0.921**	**0.831 [0.726–0.937]**
	3	2176.53	0.763	0.467 [0.301–0.632]
	4	2073.38	0.895	0.768 [0.646–0.890]
	5	1879.99	**0.921**	0.830 [0.724–0.936]
	6	1812.48	0.851	0.684 [0.546–0.822]
	7	1869.91	0.877	0.745 [0.621–0.870]
	8	1854.42	0.675	0.197 [0.061–0.332]
	Average	1989.97	0.851	

The best performing iteration for each model is highlighted in bold.

**FIGURE 4:**
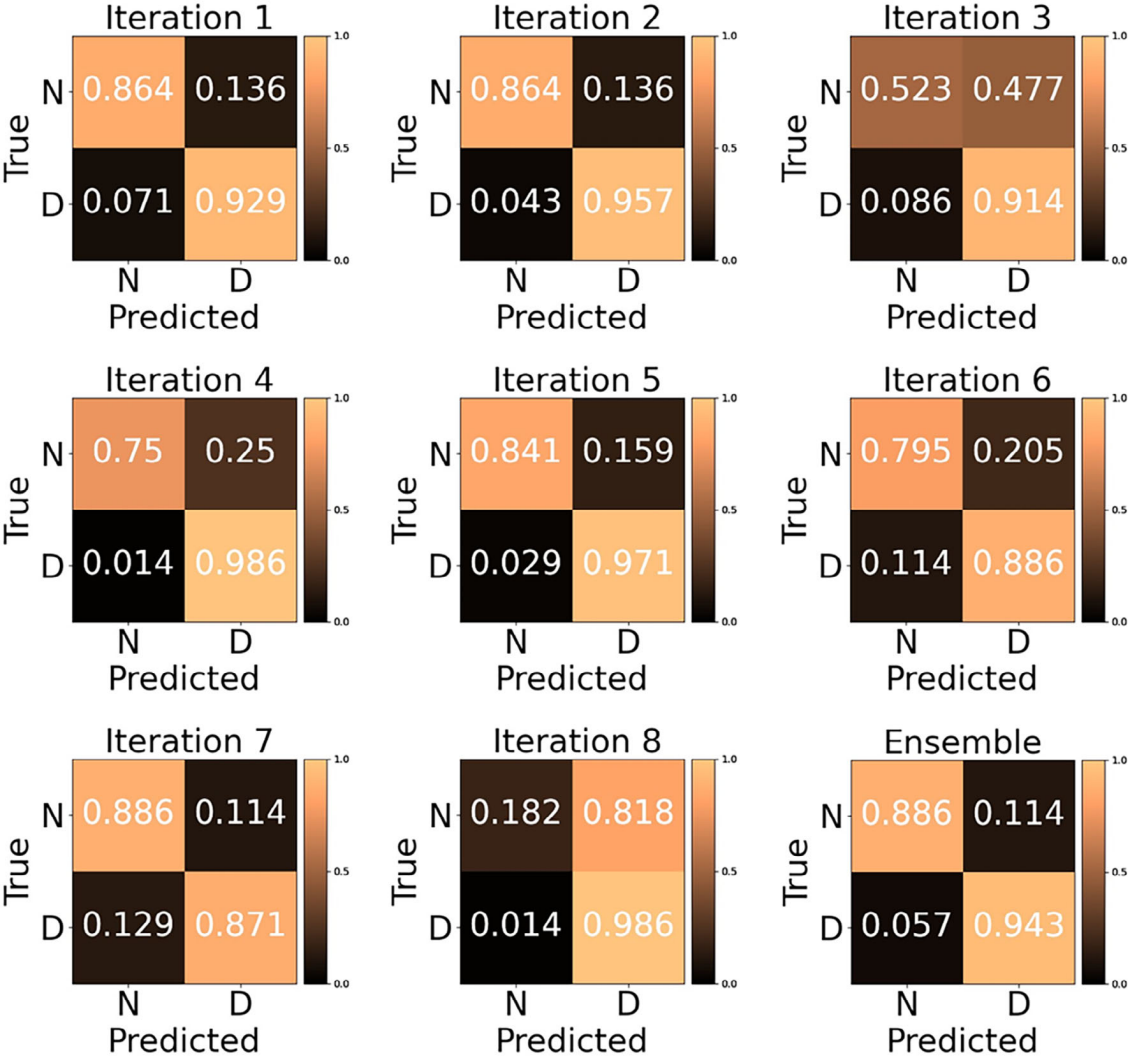
Confusion matrices for each cross‐validation iteration, where (N) signifies non‐diagnostic image quality and (D) signifies diagnostic image quality, using the test dataset (114 slices). The matrices were generated for the best‐performing architecture, SqueezeNet, as well as its corresponding ensemble decision‐making approach. The color gradient represents the % of images of each true class that were predicted to be N or D. A perfect classification would be represented by 1.0 in the main diagonal (top‐left and bottom‐right) of a confusion matrix.

**FIGURE 5:**
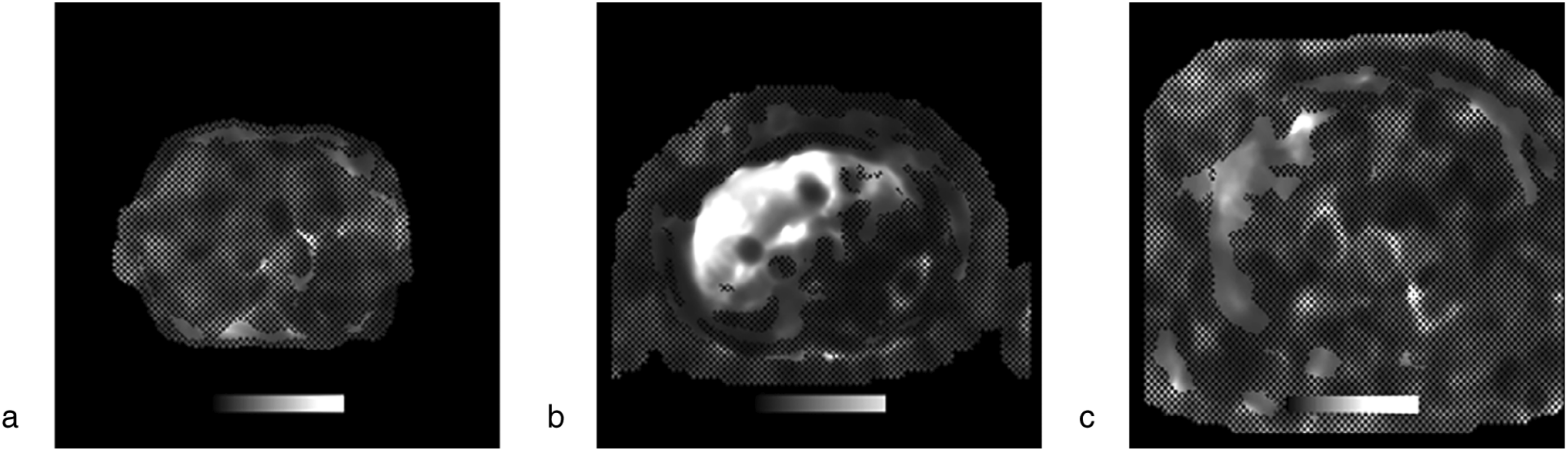
Representative CMOE slices from the test set with their reference standard quality labels and corresponding ensemble predictions. Both the observer and the ensemble classified as non‐diagnostic (**a**), both the observer and the ensemble classified as diagnostic (**b**), and the observer classified as diagnostic while the ensemble as non‐diagnostic (**c**).

Confusion matrices of each cross‐validation iteration of the SqueezeNet are included to show the class‐specific predictions versus observer labeling (Fig. 4). The ensemble decision‐making approach for the SqueezeNet achieved an accuracy of 0.921, a precision of 0.907, and a recall of 0.887 in the test set. Representative CMOE slices from the test set with their reference standard quality labels and corresponding ensemble predictions are shown in Fig. 5. Lastly, the SqueezeNet models trained in the shortest amount of time (average duration of iteration: 1989 seconds) compared to the models using other architectures (range of averages: 2564–8015 seconds) (Table 3).

### Agreement Between Models and Reference Standard

The models using the SqueezeNet architecture achieved varying levels of agreement with the reference standard, including slight (0–0.2) in one iteration, moderate (0.41–0.60) in one iteration, substantial (0.61–0.80) in four iterations, almost perfect (0.81–1.00) in two iterations and almost perfect in the ensemble decision‐making approach (Cohen's unweighted Kappa 0.833 [95% CI 0.73–0.94]) (Table 3). Among the other architectures, the highest Kappa coefficient between the reference standard and the model was achieved at the eighth iteration of the Resnet50 cross‐validation (Kappa: 0.760, accuracy: 0.886). For the lowest performing architecture, the MobileNet, the second iteration was the best iteration which yielded a Kappa of 0.393 and an accuracy of 0.728.

## Discussion

Our study assessed the performance of various established DL architectures for classifying liver MRE quality using annotated CMOE slices. We found that SqueezeNet models yielded the highest average accuracy (0.851), average precision (0.880), and average recall (0.713) in the test dataset, with a human observer's result as a reference standard. Furthermore, the average training duration across the SqueezeNet models (1989 seconds) was faster than those of the models using other architectures (range of average training times: 2564–8015 seconds).

Because our DL model can reduce the quality evaluation step to just a few seconds, it may enable technicians and/or radiologists to focus on correcting the quality issues, such as adjusting hardware and correcting patient motion, so that reacquisitions may be performed as required within the session's time constraints.

In this study, we considered each slice from an MRE exam as an input, rather than the entire dataset of the MRE exam. This approach was favorable for increasing the sample size of the dataset. In addition, slices from any repeated MREs from the same patient could be used in the dataset since different MRE in the same patient could yield different quality outcomes. To accommodate this, data from the same patients might have appeared in multiple folds during the stratified cross‐validation, as each CMOE slice was evaluated individually rather than giving a single score to a complete exam. An alternative approach would have required a 3D input, with a much larger sample size, and potentially different CNN architectures, which could be the subject of future studies. Given these considerations, we do not expect a major bias to affect the model evaluation from the potential of data from a single acquisition being present in both the training and validation cohorts.

Substantial agreement or higher was observed between the best‐performing DL model (SqueezeNet architecture) and the reference standard across six of the eight cross‐validation iterations, reaching a maximum of 0.831 in the second iteration. The Kappa of each iteration was lower than the inter‐observer agreement (Kappa 0.896), but the Kappa achieved in three different iterations surpassed the substantial inter‐observer agreement reported in a previous study by Wagner et al (Kappa = 0.781).^9^ Our approach establishes a baseline for automating MRE quality control using a well‐established CNN architecture (SqueezeNet) and achieving an average test accuracy of 0.851, with a peak test accuracy of 0.921 in its best‐performing iteration.

Comparing the performance of five CNN architectures (ResNet18, ResNet34, ResNet50, SqueezeNet, and MobileNetV2), it is noteworthy that despite having a lower count of trainable parameters, SqueezeNet exhibited the best performance in our study. Specifically, SqueezeNet achieved superior results in terms of accuracy, recall, and Cohen's unweighted Kappa coefficient for the test dataset compared to the other architectures upon training. The superior performance of SqueezeNet despite its lower count of trainable parameters could be attributed to its unique architecture. SqueezeNet introduces several innovative design choices, such as replacing the traditional large filter size with a combination of 1 × 1 and 3 × 3 filters, which significantly reduces the number of parameters without compromising performance. Furthermore, the lower number of trainable parameters (0.7M) of the SqueezeNet is likely to be more favorable for the smaller scale dataset used in this study. Yet, a lightweight architecture does not necessarily guarantee good performance as demonstrated by the MobileNetV2 (2.2M trainable parameters) which yielded the lowest average test set accuracy (0.692) of the tested architectures.

### Limitations

Our study has several limitations. First, the CNN models tested do not provide information about the root cause of the failed MRE acquisition, which would be valuable for practical decision‐making, such as fixing hardware issues and repeating imaging. However, training such a model would require a larger dataset with a sufficient number of samples for each failure cause. Second, during the CMOE labeling process, observers could inspect all associated MRE output image types, as is done in the clinical setting to determine quality. Our models were trained using only CMOEs for the classification of the generalized acquisition quality. Future studies would include other MRE images (eg, phase images, magnitude images, etc.) generated during the acquisition process in a multi‐input multi‐channel classification model. This is expected to improve model performance by identifying failure causes that are visible in the other image types. Since visual information within CMOE slices is unlikely to capture all the failure causes, studying the optimal combination of MRE image types needed for high‐accuracy DL‐based quality control is warranted. Third, despite a high inter‐observer Kappa value, it should be noted that both observers have limited MRE experience in abdominal imaging. In addition, we relied on a single observer for the reference standard, rather than the consensus of multiple observers or experienced experts. Lastly, the curated dataset comprised of a single vendor, potentially limiting the application of the presented models to other vendors.

## Conclusion

In conclusion, an automated DL‐based classification of liver 2D MRE diagnostic quality demonstrated promising performance, in which the highest performing models (SqueezeNet architecture) achieved an average accuracy of 0.851 (range 0.675–0.921) in the test dataset.
